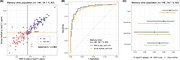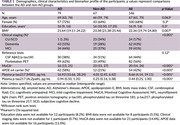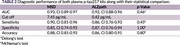# Head to head comparison of plasma phosphorylated tau 217 assays in real life memory clinic in Thailand

**DOI:** 10.1002/alz70856_104726

**Published:** 2026-01-07

**Authors:** Watayuth Luechaipanit, Thanaporn Haethaisong, Adipa Chongsuksantikul, Prawit Oangkhana, Kittithatch Booncharoen, Jedsada Khieukhajee, Yuttachai Likitjaroen, Poosanu Thanapornsangsuth

**Affiliations:** ^1^ Thai Red Cross Emerging Infectious Diseases Health Science Centre, King Chulalongkorn Memorial Hospital, Bangkok, Thailand; ^2^ Thai Red Cross Emerging Infectious Diseases Health Science Centre, King Chulalongkorn Memorial Hospital, The Thai Red Cross Society, Bangkok, Thailand; ^3^ Neurology Center, Phyathai 1 Hospital, Bangkok, Rachathewi, Thailand; ^4^ Neurocognitive Unit, Division of Neurology, Department of Medicine, Faculty of Medicine, Chulalongkorn University, Bangkok, Thailand; ^5^ Memory Clinic, King Chulalongkorn Memorial Hospital, The Thai Red Cross Society, Bangkok, Thailand; ^6^ Neurological Institute of Thailand, Ratchathewi, Bangkok, Thailand; ^7^ Division of Neurology, Department of Medicine, Faculty of Medicine, Chulalongkorn University, Bangkok, Thailand

## Abstract

**Background:**

Recently, immunoassay‐based plasma phosphorylated tau 217 (*p*‐tau217) was commercially available as research used only (RUO) test kit. Due to its exceptional performance compared to other *p*‐tau species and designation as a core early‐changing biomarker, *p*‐tau217 may become a standalone for Alzheimer's disease biomarker, potentially gaining regulatory approval in the near future. However, not every immunoassay demonstrates high diagnostic accuracy in clinical setting. In this study, we retrospectively evaluated 146 participants, comparing the performance of single molecule assay (Simoa) on SR‐X platform and electrochemiluminescence (ECL) on Meso Scale Discovery (MSD) Quickplex SQ120 platform.

**Method:**

Participants with cognitive complaints were enrolled between 2022 and October 2024 during clinical visits at the Memory Clinic at King Chulalongkorn Memorial Hospital and Neurology Clinic at Neurological Institute of Thailand. A head‐to‐head comparison of commercially available of immunoassay‐based plasma *p*‐tau217 quantification was conducted on 146 samples. Amyloid‐positron emission tomography (PET) using [^18^F]‐Florbetaben or CSF amyloid‐β42/p‐tau were performed as reference tests to distinguish AD and non‐AD patients. Optimal cut‐offs were defined using the Youden's Index. Diagnostic performance of both plasma *p*‐tau217 kits was statistically compared using Delong's test, McNemar Test.

**Result:**

Among 146 participants enrolled, 100 (68%) were female. The median age was 67 years. With 79 participants (54.1%) were confirmed to have AD. Plasma *p*‐tau217 (MSD) had an area under the curve (AUC) of 0.932 (95% confidence interval (CI) 0.89‐0.97) with 90% sensitivity and 85% specificity, while plasma *p*‐tau217 (Simoa) had AUC of 0.919 (CI 0.88‐0.96) with 86% sensitivity and 87% specificity for distinguishing AD and non‐AD patients. Single cut‐point value was evaluated based on Youden index as < 7.45 pg/mL for MSD and < 0.42 pg/mL for Simoa SR‐X. Differences are not significant.

**Conclusion:**

The diagnostic performance of both immunoassay‐based plasma *p*‐tau217 was shown equivalent to benchmark tests, also accurately diagnosing AD among participants enrolled from specialized memory clinic. Utilizing plasma *p*‐tau217 biomarker may serve as an accurate AD diagnostic tool in real‐world memory clinic.